# First person – Aitana Manuela Castro Colabianchi

**DOI:** 10.1242/bio.058656

**Published:** 2021-03-02

## Abstract

First Person is a series of interviews with the first authors of a selection of papers published in Biology Open, helping early-career researchers promote themselves alongside their papers. Aitana Manuela Castro Colabianchi is first author on ‘Segregation of brain and organizer precursors is differentially regulated by Nodal signaling at blastula stage’, published in BiO. Aitana Manuela conducted the research described in this article while a PhD student in Dr Silvia Liliana Lopez's lab at the Institute for Cell Biology and Neuroscience, University of Buenos Aires. She is now a postdoc in the lab of Dr Lucía Florencia Franchini at INGEBI, 2490 Vuelta de Obligado, Buenos Aires, investigating those mechanisms that first metazoans developed and still have impact in our lives.


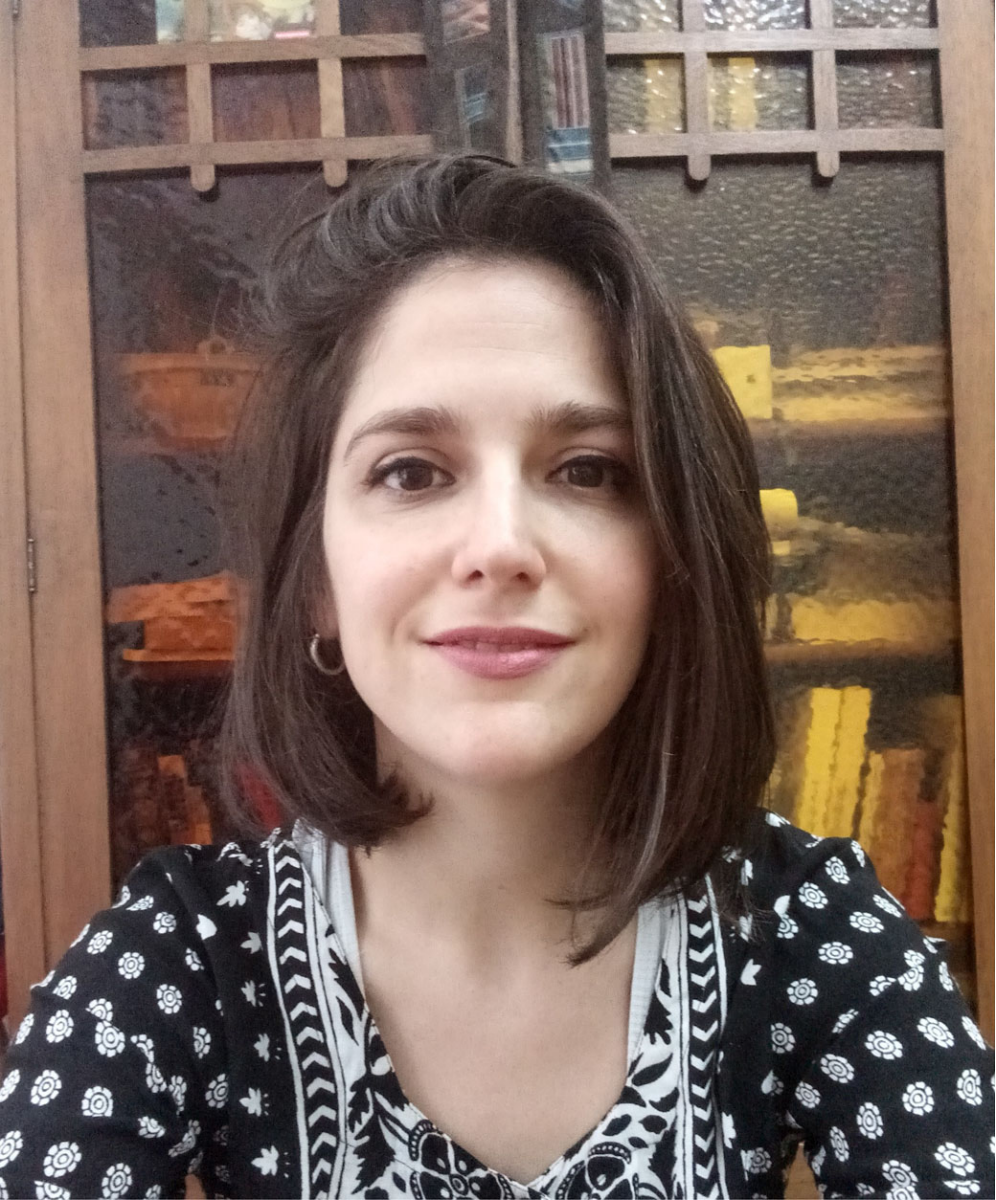


**Aitana Manuela Castro Colabianchi**

**What is your scientific background and the general focus of your lab?**

I am a biologist. I studied Biology BS/MS and Biology Education BEd simultaneously at National University of Cordoba. Throughout those years I got involved in a variety of research projects from very different disciplines such as environmental biology, biophysics, developmental genetics, statistics, etc.

In order to complete my bachelor thesis in the field of developmental biology, I moved to the Dr Andres Carrasco Laboratory, currently led by Dr Silvia Lopez, to study the role of *foxa4* in the dorsal midline of *Xenopus*. Thereafter I started my PhD at the same laboratory where I contributed to the understanding of the mechanisms by which Notch1 can control dorsal–ventral axis development. The lab continues to study the roles of Nodal and Notch1 canonical, and non-canonical pathways on vertebrates' body plan. Right now I am working on a short postdoctoral project about the role of npas3 during early development at Dr Lucía Franchini Laboratory.

**How would you explain the main findings of your paper to non-scientific family and friends?**

During early *Xenopus* (frog) development the embryo looks like a sphere called the ‘blastula’. The blastula is made up of cells that look the same. A group of these cells expresses a gene called *chordin*, and they will differentiate during development until giving rise to some of the precursors of the brain and spine among others. Considering that these *chordin-*expressing cells look the same, it was assumed that all of them had the same potential to be part of either the brain or the spine.

We manipulated the activity of Nodal protein in *Xenopus* embryos, and we found that a subpopulation that normally expresses *chordin* stopped doing so (green asterisk), while another subpopulation kept its expression intact (above green asterisk). Following the specialized markers of these cells, we saw that the subpopulation that lacked chordin was the one that contributed to the spine.

In sum the cells that express *chordin* at blastula stage look the same, because they are receiving the same message to behave that way. But that message is carried by different messengers, so it matters who the message comes from because if one of them is silent (such as Nodal) the cells lacking the message stop expressing *chordin*, and they cannot be what they were supposed to be anymore.

We were able to show that it is possible to recognize as early as the blastula stage, the cells that will contribute to forming the brain and those that will contribute to forming the spine.

**What are the potential implications of these results for your field of research?**

Our experiments have been done in *Xenopus*, we also compared them with the results obtained by others on mice, zebrafish, and chicken. Everything suggests that the decisions about the cell sinks that determine organ size and midline structures development begin before gastrulation in all vertebrates analysed. This means that the developmental pathways involved in this process and the particular study of the dorso-ventral development and the organs related to it (brain, spinal cord, the spine, and others) should broaden their focus towards earlier stages of development.

**What has surprised you the most while conducting your research?**

We were able to hypothesize a developmental process through a spatial analysis of an embryo processed by *in situ* hybridization technique (ISH). Also, in our previous work about the role of *notch1* in dorsal-ventral axis development, we found a non-uniform bmp4 expression within notch1 depleted siblings analysed by ISH. This sub-populations’ variability might be the snapshots of an oscillatory behaviour. In such cases, complex results might become invisible using quantitative techniques such as RT-qPCR, because they retrieve mean values rather than descriptive information about the existing variability between cells and embryos. Although time continues to pass, it is surprising for me the enormous explanatory power that ISH has.

**Figure:**
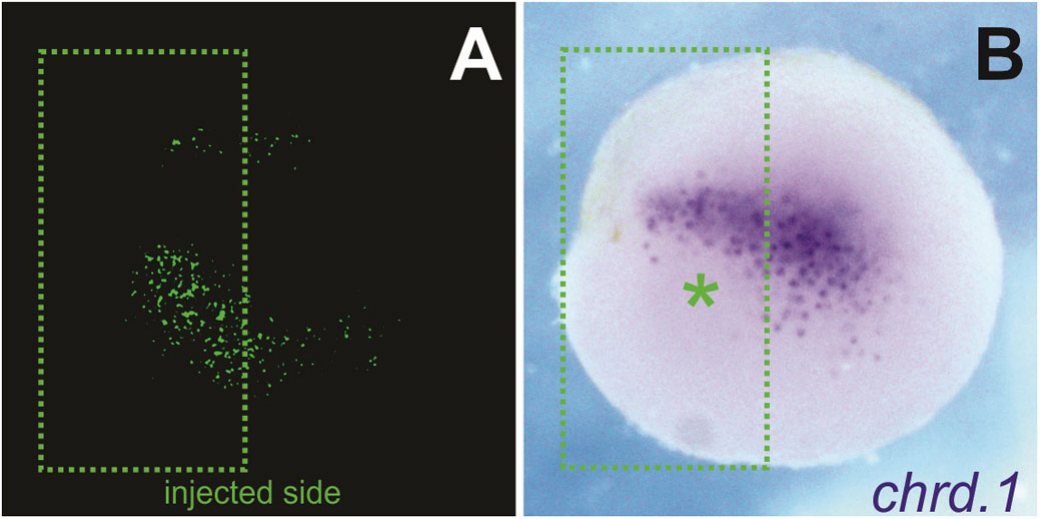
**A dual behaviour of *chordin* expression is observed through the injected side of a *Xenopus* embryo (Nodal blocked side: green dashed squares).**

“Although time continues to pass, it is surprising for me the enormous explanatory power that ISH has.”

**What changes do you think could improve the professional lives of early-career scientists?**

First, focusing on goals rather than disciplines, ecology brings you the perspective of populations, and as we know cell populations are also variable, Philosophy can make you understand that the causes you claim in your results are not other than consequences. Even feminist reading can help us to think about the preconceptions that shape the hypotheses we formulate. Therefore, taking a wide spectrum of courses and reading widely will provide us with a broader view of our research problems, and take our career to a higher level.

Second, the early career time is when we are being exposed to a big amount of information from courses, papers and ideas from our peers. All this effort should be translated into scientific production, such as review articles, the organization of scientific events, and project formulation. In recent years, there have been more opportunities for students to be involved in the full scope of a PI, such as research grants and workshops for students and the possibility to publish our review articles while we are doctoral students. We must take the challenge.

**What's next for you?**

I am joining Dr Sheng Zhong's laboratory at UCSD, to study the role of RNAs in tissue recognition at the ultra-cellular level. I will also work on a research project about the role of RNAs on inter-individual communication at inter and intraspecific levels. I am also preparing a review proposal to summarize the roles that RNAs have been given during the last 60 years according to molecular biology and molecular evolution perspectives.
